# Aging and neuroinflammation: Changes in immune cell responses, axon integrity, and motor function in a viral model of progressive multiple sclerosis

**DOI:** 10.1111/acel.13440

**Published:** 2021-08-06

**Authors:** Leyre Mestre, Graciela Alonso, Ana Feliú, Miriam Mecha, Carolina Martín, Luisa M. Villar, Carmen Guaza

**Affiliations:** ^1^ Neuroimmunology Group Functional and Systems Neurobiology Department Instituto Cajal CSIC Madrid Spain; ^2^ Red Española de Esclerosis Múltiple (REEM) Barcelona Spain; ^3^ Immunology Department Hospital Universitario Ramón y Cajal Instituto Ramón y Cajal de Investigación Sanitaria (IRYCIS) Madrid Spain

**Keywords:** aging, cellular innate and adaptive immunity, neuroinflammation, progressive multiple sclerosis, Theiler's virus

## Abstract

Although aggravated multiple sclerosis (MS) disability has been reported in aged patients, the aging impact on immune cells remodeling within the CNS is not well understood. Here, we investigated the influence of aging on immune cells and the neuroinflammatory and neurodegenerative processes that occur in a well‐established viral model of progressive MS. We found an anomalous presence of CD4^+^ T, CD8^+^T, B cells, and cells of myeloid lineage in the CNS of old sham mice whereas a blunted cellular innate and adaptive immune response was observed in Theiler's murine encephalomyelitis virus (TMEV) infected old mice. Microglia and macrophages show opposite CNS viral responses regarding cell counts in the old mice. Furthermore, enhanced expression of Programmed Death‐ligand 1 (PD‐L1) was found in microglia isolated from old TMEV‐infected mice and not in isolated CNS macrophages. Immunocytochemical staining of microglial cells confirms the above differences between young and old mice. Age‐related axonal loss integrity in the mouse spinal cord was found in TMEV mice, but a less marked neurodegenerative process was present in old sham mice compared with young sham mice. TMEV and sham old mice also display alterations in innate and adaptive immunity in the spleen compared to the young mice. Our study supports the need of new or adapted pharmacological strategies for MS elderly patients.

## Graphical abstract

A review of CNS immune changes of Theiler's virus‐infected mice at young (12 weeks after infection) and old age (40 weeks after infection). The type of changes in the response to viral infection results of the comparison with the corresponding changes observed in young and old sham mice. The age‐associated immune dysfunction may reduce the ability of the immune system to respond efficiently to the pathological consequences induced by Theiler's murine encephalomyelitis virus during the later chronic phases of the disease.

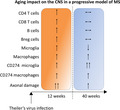

## 1 INTRODUCTION

Multiple sclerosis (MS) is a chronic inflammatory demyelinating CNS disease characterized by reversible neurological deficits associated with inflammation and secondary progressive neurodegeneration. About 80% of MS patients are diagnosed with the relapsing‐remitting form (RRMS), but 70% of these patients shift to a progressive disease course (PMS) as they age (Tutuncú et al., 2013). This change has been related to immunosenescence, the changes that occur in the cellular immune response as a result of aging. Indeed, premature immune aging has been described in some MS patients, perhaps underlying the transition from the RR form to a progressive disease course (Thewissen et al., 2005). In this context, it is also significant that most MS patients are diagnosed between 20 and 40 years of age, and only a few individuals are diagnosed after they have reached 50 years of age (Scalfari et al., 2014). In recent years, interest in understanding the impact of aging on the course of MS has emerged in an attempt to find novel therapies or to adapt current treatments to the chronological age of patients (Vaughn et al., 2019).

Key age‐related changes in the CNS are triggered by microglia, yet a diminished efficiency of the adaptive and innate immune cell responses may be contributing to the pathological progression of the disease in elderly MS patients (Bektas et al., 2017; Musella et al., 2018; Rawji et al., 2016). Exaggerated immunosenescence is a concept that has been employed in MS trying to explain the accelerated changes in remodeling immune response during aging (Mandolesi et al., 2015). Although it has been postulated that the lesions that develop in aging patients may do so on a pre‐injured CNS, it is not clear whether this may also be the case in experimental models (Musella et al., 2018; Wimmer et al., 2019).

Most experimental studies on aging impact in MS have been performed by modeling the RRMS course following immunization with myelin proteins (Bolton & Smith, 2018), yet it remains unknown what happens in experimental progressive MS. One of the most applicable models to study how age affects the course of progressive MS is the Theiler's murine encephalomyelitis virus‐induced demyelinating disease (TMEV‐IDD) mode as this is the reference model to investigate viral‐mediated mechanisms of primary progressive MS (Olson et al., 2001). Intracerebral infection with TMEV in susceptible mice induces a chronic progressive neurodegenerative disease that is clinically and histopathologically similar to human progressive MS (Denic et al., 2011; Lipton & Dal Canto, 1976). Here, we focused on CNS and spleen cellular immune responses during the neurodegenerative phase of the disease, by assessing axonal damage and motor disability, both well reproduced in the TMEV model and that potentially reflect the pathological changes in the progressive phase of MS. Hence, the aim of our study was to investigate the impact of aging on neuroinflammation particularly, in microglia and macrophage cells that play a critical role in the pathogenesis of MS. Age‐related alterations in the expression of the immune checkpoint PDL‐1 by microglia and macrophages together with the inability to mount an adequate cell innate and adaptive responses during TMEV infection may be affecting the neurodegeneration and disability in TMEV‐IDD.

## 2 RESULTS

### 2.1 Aging diminishes CD4^+^ and CD8^+^ T‐cell immune responses in the CNS of TMEV‐infected mice

**FIGURE 1:**
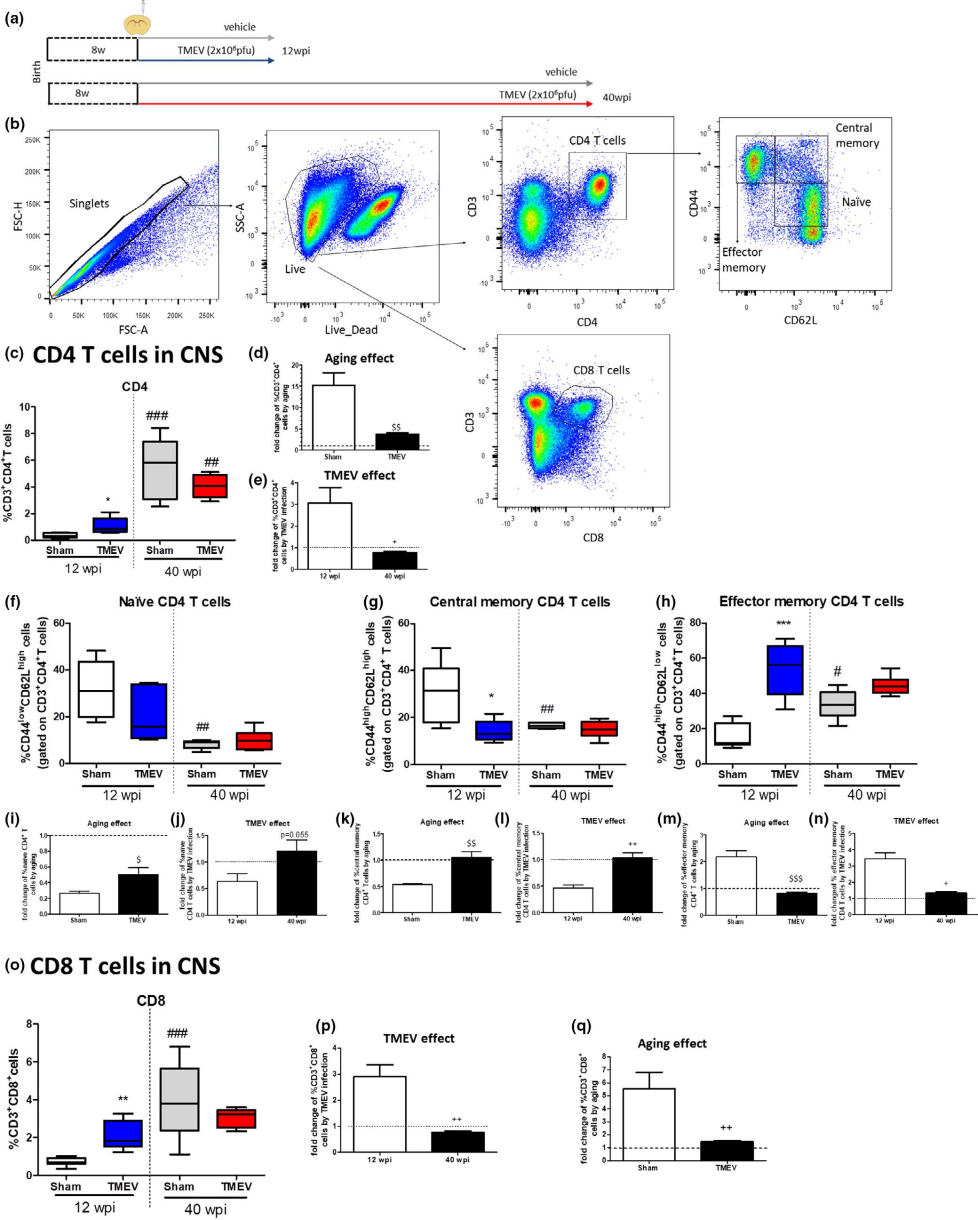
Aging impairs the CD4^+^ and CD8^+^ T‐cell immune responses in the CNS of Theiler's murine encephalomyelitis virus (TMEV)‐infected mice. (a) Scheme of the experimental groups, where 8‐week‐old mice were infected with 2 × 10^6^ pfu of Theiler's virus and followed for 12 or 40 weeks. (b) Representative FACs plots of the gating strategy used to identify the different T‐cell populations. (c) Quantification of the CD3^+^CD4^+^ T cells in CNS. (d, e). Fold change of relative percentage of CD4^+^ T cells induced by aging and TMEV, respectively (f–h) Quantification of the percentage of naïve, central memory and effector memory CD4^+^ T cells, together with the change induced by aging (i, k, m) or TMEV infection (j, l, n), respectively. (o) Quantification of CD3^+^CD8^+^ T cells in CNS. (p, q) Fold change of the relative percentage of CD3^+^CD8^+^ T cells induced by aging or TMEV infection, respectively. The data are presented as the mean ±SEM: *p < 0.05 vs. sham at the same age; **p < 0.01 vs. sham at the same age; ^#^ *p* < 0.05 vs. the same experimental group at 12 wpi; ^##^ *p* < 0.01 vs. the same experimental group at 12 wpi; ^###^ *p* < 0.001 vs. the same experimental group at 12 wpi; ^+^ *p* < 0.05 vs. 12 wpi; ^++^ *p* < 0.01 vs. 12 wpi; ^$^ *p* < 0.05 vs.sham; ^$$^ *p* < 0.01 vs. sham; ^$$$^ *p* < 0.001 vs. sham (*n* = 6 sham 12 wpi; *n* = 6 TMEV 12 wpi; *n* = 5 sham 40 wpi; *n* = 5 TMEV 40 wpi)

The course of TMEV‐IDD infection in SJL/J mice indicates that by 12 wpi, animals develop motor deficits and CNS neuroimmune alterations as we previously described (Mecha et al., 2013). However, it is unknown how aging affects the adaptive immune responses of TMEV‐infected mice. In order to investigate this, we infected 8‐week‐old female mice with TMEV (Figure 1a) and 12 or 40 weeks later, we analyzed the T‐cell populations following the gating strategy shown in Figure 1b. Note that the mice of the SJL/J strain experience physiological age‐related events before other mouse strains like C57BL/6 or BALB (Murray et al., 1998), with SJL/J mice having a shorter lifespan. TMEV infection increased the frequency of CD4^+^ T cells in the CNS at 12 (*p* < 0.05) but not at 40 wpi (Figure 1c). This surprising result is partially due to the fact that aging increases the proportion of CD4^+^ T cells (*p* < 0.001 vs. 12 wpi Sham and *p* < 0.01 vs. 12 wpi TMEV; Figure 1c), being significantly higher in sham mice than in TMEV‐infected mice (*p* < 0.01 vs. Sham; Figure 1d). Therefore, the relative change in the proportion of CD4^+^ T cells induced by TMEV was significantly different between young and old animals (*p* < 0.05 vs 12 wpi TMEV; Figure 1e). Aging significantly diminished the naïve and central memory T cells, while there were more effector memory CD4^+^ T cells in old sham mice (*p* < 0.05 vs 12 young sham mice; Figure 1f–h). Specifically, the decrease of both naïve and central memory was significantly greater in sham mice than in TMEV mice (Figure 1i,k). In addition, while aging doubled the effector memory cells population in sham mice, this was practically not unchanged in infected mice (Figure 1m). Regarding the effect of the TMEV infection, the proportion of central and effector memory CD4^+^ T cells were decreased and augmented, respectively, in young mice, but not in old mice (Figure 1g,h). This means that while infection with TMEV induces a decrease in naïve and central memory cell populations in young mice (Figure 1j,l), effector memory CD4 T cells were significantly increased (Figure 1n). However, in the case of old mice, TMEV infection did not significantly modify these immune populations, except for a tendency to increase the population of naïve cells (Figure 1j,l,n). Collectively, our results not only demonstrate the anomalous presence of CD4^+^ T cells in old sham mice but also show a deficient age‐related CD4^+^ T cells response in the CNS of mice subjected to TMEV infection as a model of progressive MS.

CD8^+^ T cells showed a similar profile to CD4^+^ T cells. As such, CD8^+^T cells were increased in response to TMEV in the young mice only (*p* < 0.01 vs. 12 wpi sham: Figure 1o). Again, old sham mice have an elevated frequency of CD8^+^ T cells (*p* < 0.001 vs. 12 wpi sham: Figure 1o,p); however, TMEV‐infected mice did not show this increase (Figure 1p). Notably, there was a weaker relative fold change in the CD8^+^ T‐cell response in the CNS of TMEV 40 wpi mice (*p* < 0.01 vs. TMEV 12 wpi: Figure 1q), which may lead to a deficient control of inflammation in the aged brain. As such, the age of the mice clearly affects the CNS cellular adaptive responses in TMEV‐IDD mice.

### 2.2 B‐cell responses to long‐term TMEV infection are limited in aging mice

**FIGURE 2:**
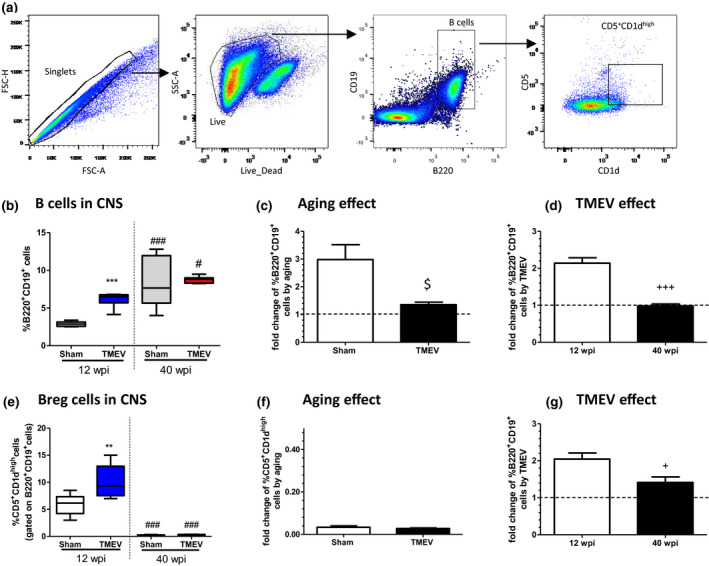
B‐cell responses to long time Theiler's murine encephalomyelitis virus (TMEV) infection were limited in aging mice. (a) Representative flow cytometry plots of the gating strategy. (b) Quantification of the percentage of B220^+^CD19^+^ B cells in the CNS; (c, d) Fold change of relative percentage of B220^+^CD19^+^ cells aging‐ or TMEV‐induced, respectively (e) Quantification of the proportion of CD5^+^CD1d^high^ cells gated on B220^+^CD19^+^ B cells. (f, g) Fold change of relative percentage of CD5^+^CD1d^high^ cells induced by aging or TMEV, respectively The data are expressed as the mean ± SEM (*n* = 5–6 mice for each group): **p < 0.01 vs. sham at the same age; ***p < 0.001 vs. sham at the same age; ^###^ *p* < 0.001 vs. the same experimental group at 12 wpi; ^$^ *p* < 0.05 vs. sham (*n* = 6 sham 12 wpi; *n* = 6 TMEV 12 wpi; *n* = 5 sham 40 wpi; *n* = 5 TMEV 40 wpi) +*p* < 0.05 vs. 12 wpi and +++*p*<0.001 vs. 12wpi

The involvement of the B‐cell population in MS pathogenesis is undeniable (Sospedra, 2018) and clear shifts in B cells in the elderly have been described, suggesting that age‐related B‐cell changes contribute to immunosenescence (Hao et al., 2011). Next, we studied B cells by FACs in the model of progressive MS (Figure 2a). A higher proportion of B cells was detected in the CNS of young TMEV mice relative to control sham animals (*p* < 0.001 vs 12 wpi sham mice). However, this effect was not evident at 40 wpi (Figure 2b) because as observed with T cells, aging per se augmented the proportion of B cells in the CNS of old sham mice (*p* < 0.001 vs. 12 wpi sham mice). Despite this apparent lack of change in B‐cell population in old mice due to TMEV infection, it must be taken into account that the increase of B cells produced by aging is significantly greater in sham mice than in TMEV mice (*p* < 0.05 vs. Sham). Consequently, while infection with TMEV doubles the proportion of B cells in young mice, it was not significantly modified in old mice (Figure 2d). Therefore, a CNS blunted response was driven also in B cells in old TMEV mice. We next examined the frequency of CD5^+^CD1d^high^ B cells involved in the regulation of immune responses. TMEV infection induced an increase in Breg cells in the CNS of young mice (*p* < 0.01 vs. 12 wpi sham: Figure 2e) but not in old mice, being significant the discrepancy of effect between young and old mice by TMEV infection (Figure 2g). In contrast, there was a significant age‐dependent reduction in the percentage of CD5^+^CD1^high^ Breg cells (*p* < 0.001 vs. young sham and *p* < 0.001 vs. young TMEV mice). The fold decrease was similar in both sham and TMEV mice (Figure 2f).

### 2.3 Aging impact microglia and macrophages in an opposite way in the CNS of TMEV‐infected mice

**FIGURE 3:**
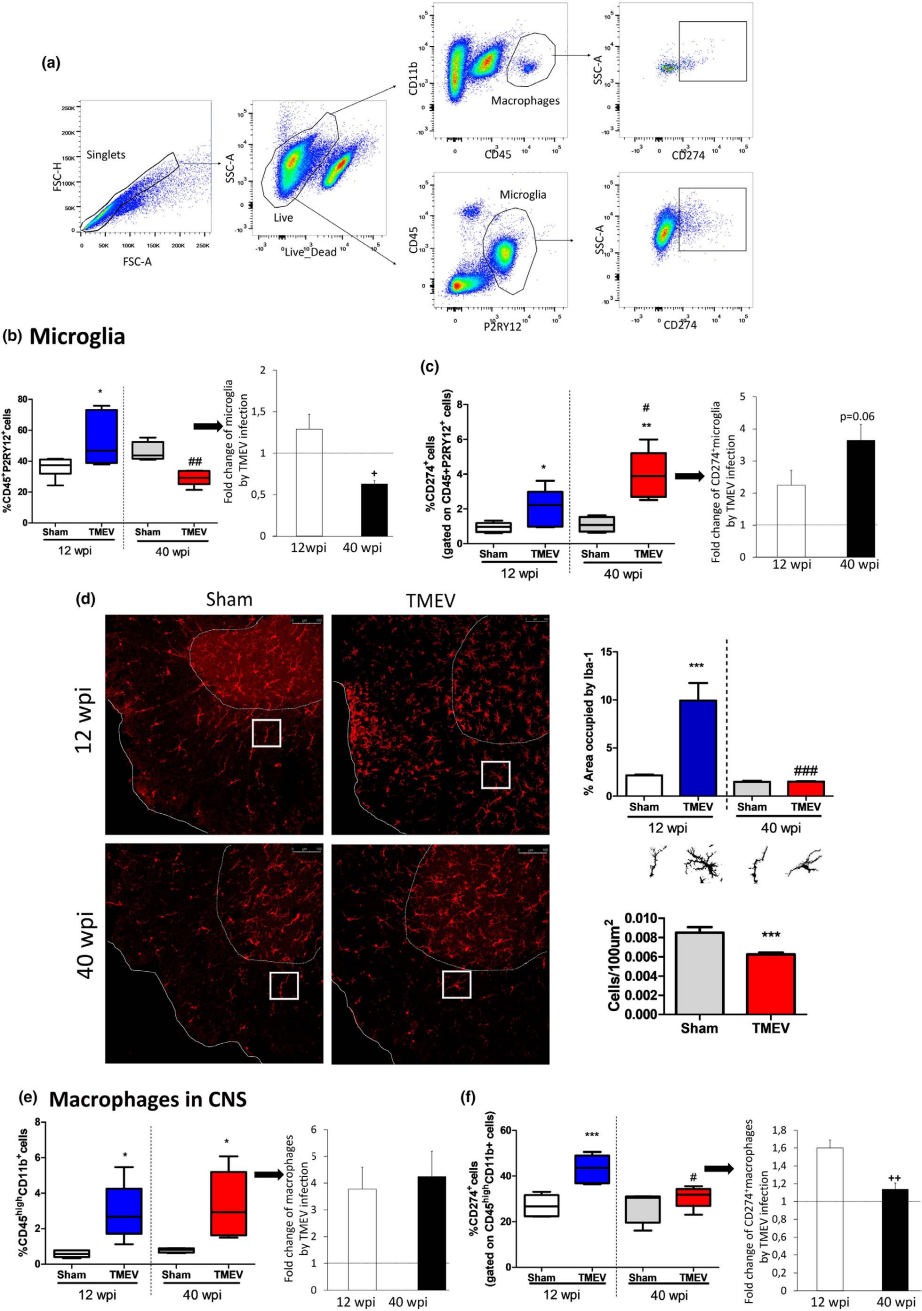
Aging alters microglia and macrophages distinctly in the CNS of Theiler's murine encephalomyelitis virus (TMEV)‐infected mice. We used the purinergic receptor P2RY12 as a marker of homeostatic microglia and CD45^high^CD11b^+^ labeling for macrophages. (a) Representative FACs plots showing the gating strategy to identify CD274^+^ macrophages or microglia.(b) Quantification of the percentage of CD45^+^P2RY12^+^ cells together with the change in microglia induced by TMEV infection at 12 or 40 wpi. (c) Quantification of the percentage of CD274+ cells gated on CD45^+^P2RY12^+^ cells together with the change in these cells induced by TMEV infection at 12 or 40 wpi. (d) Representative images of transverse spinal cord sections immunostained with the Iba‐1 antibody. Scale bar 100 μm. These images are accompanied by the quantification of the relative area occupied by Iba‐1 staining in the white matter of ventral spinal cord, at cervical level together a representative image of the cell morphology of each experimental group and quantification of the number of Iba‐1^+^cells/100 μm^2^. (e) Quantification of the proportion of CD45^high^CD11b^+^ cells in the CNS together with the change in macrophages induced by TMEV infection at 12 and 40 wpi. (f) The percentage of CD274^+^ cells gated on CD45^high^CD11b^+^ cells is shown, along with the change in these cells induced by TMEV infection at 12 and 40 wpi. The data are expressed as the means ± SEM: *p < 0.05 vs. sham at the same age; **p < 0.01 vs. sham at the same age; ***p < 0.001 vs. sham at the same age; ^#^ *p* < 0.05 vs. the same experimental group at 12 wpi; ^##^ *p* < 0.01 vs. the same experimental group at 12 wpi; ^###^ *p* < 0.01 vs. the same experimental group at 12 wpi; +*p* < 0.05 vs. 12 wpi; ^++^ *p* < 0.01 vs. 12 wpi (*n* = 6 sham 12 wpi; *n* = 6 TMEV 12 wpi; *n* = 5 sham 40 wpi; *n* = 5 TMEV 40 wpi). FACs: *n* = 6 sham 12 wpi; *n* = 6 TMEV 12 wpi; *n* = 5 sham 40 wpi; *n* = 5 TMEV 40 wpi. Immunohistochemistry:

As CNS sensors microglia play a crucial role in health and in pathological conditions, including MS (Colonna & Butovsky, 2017). Microglial activity depends on different variables, which include age, neuropathological status, disease stage, and environmental factors (Wolf et al., 2017). In this study, we assessed the percentage of homeostatic microglia by FACs, labeling them for the markers CD45 and P2RY12 (see gating strategy in Figure 3a). Because the expression of P2RY12 can be modified by the activation state of microglia and then could be confused as macrophages, we also followed the gating strategy that try to distinguish microglia and macrophages by CD45 and CD11b markers (the population with lower CD45 expression corresponds to microglia and the population with higher CD45 expression refers to macrophages). The results observed gating on CD45^+^P2RY12^+^ cells and CD45^low^CD11b^+^ cells were similar (data not shown). The proportion of homeostatic microglia increased 12 weeks after TMEV infection compared to that found in sham mice (*p* < 0.05: Figure 3b). By contrast, the proportion of CD45^+^P2RY12^+^cells dropped in old TMEV 40 wpi mice (*p* < 0.01 vs. 12wpi TMEV: Figure 3b). Importantly, old sham mice display a proportion of microglia similar to that observed in young TMEV‐infected mice, suggesting the existence of a basal age‐related CNS inflammation in the older sham mice at least, in terms of the microglial cells count. This scenario leads to a smaller relative fold change in microglial frequency induced by TMEV in the older mice (*p* < 0.05 vs 12 wpi TMEV mice: Figure 3B). More interestingly, the proportion of microglia expressing the immune inhibitory checkpoint protein PDL‐1 (CD274) was augmented in both young and old TMEV mice in comparison with their corresponding sham mice (*p* < 0.05 and *p* < 0.01, respectively: Figure 3c). Besides, old TMEV‐infected mice had a higher proportion of microglia expressing PDL‐1 (CD274) relative to young TMEV mice 12 wpi (*p* < 0.05: Figure 3c). These results were confirmed when we analyzed the relative fold change in PDL‐1 expressing microglia. The increase of the percentage of CD274^+^ microglial cells tends to be higher in old TMEV mice respect to young TMEV mice (*p* = 0.06: Figure 3c). Immunocytochemical staining of microglial cells with Iba‐1 (Figure 3d) reveals a classic microglia response in white matter of ventral spinal cord at cervical level with a larger area occupied by Iba‐1 in 12 wpi TMEV mice (*p* < 0.001 vs. sham 12 wpi: Figure 3d), as can be visualized by the morphology of cells which was missing in 40 wpi TMEV mice (*p* < 0.001, 40 vs. TMEV 12 wpi: Figure 3d). Also there were fewer positive cells for Iba‐1 in 40 wpi TMEV mice than in 40 wpi sham mice (*p* < 0.001).

The age‐related response of CNS macrophages contrasted with that of microglia. The percentage of macrophages labeled with CD45^high^ and CD11b^+^ (see gating strategy in Figure 3a) increased similarly in the CNS of young and old mice after TMEV infection (*p* < 0.05: Figure 3e). However, the proportion of macrophages expressing PDL‐1 (CD274) only augmented in young TMEV mice 12 wpi and not after 40 weeks (*p* < 0.001 vs. 12 wpi sham mice: Figure 3e). In fact, while TMEV infection increased the proportion of CD274^+^ macrophages 1.5‐fold after 12 weeks, no such change was observed at 40 wpi (*p* < 0.01 vs TMEV 12 wpi Figure 3f). These findings suggest that age‐related regulatory mechanisms in the CNS differ between microglia and macrophages in the progressive model of MS used in our study.

### 2.4 Age‐related axonal damage in the mouse spinal cord

**FIGURE 4:**
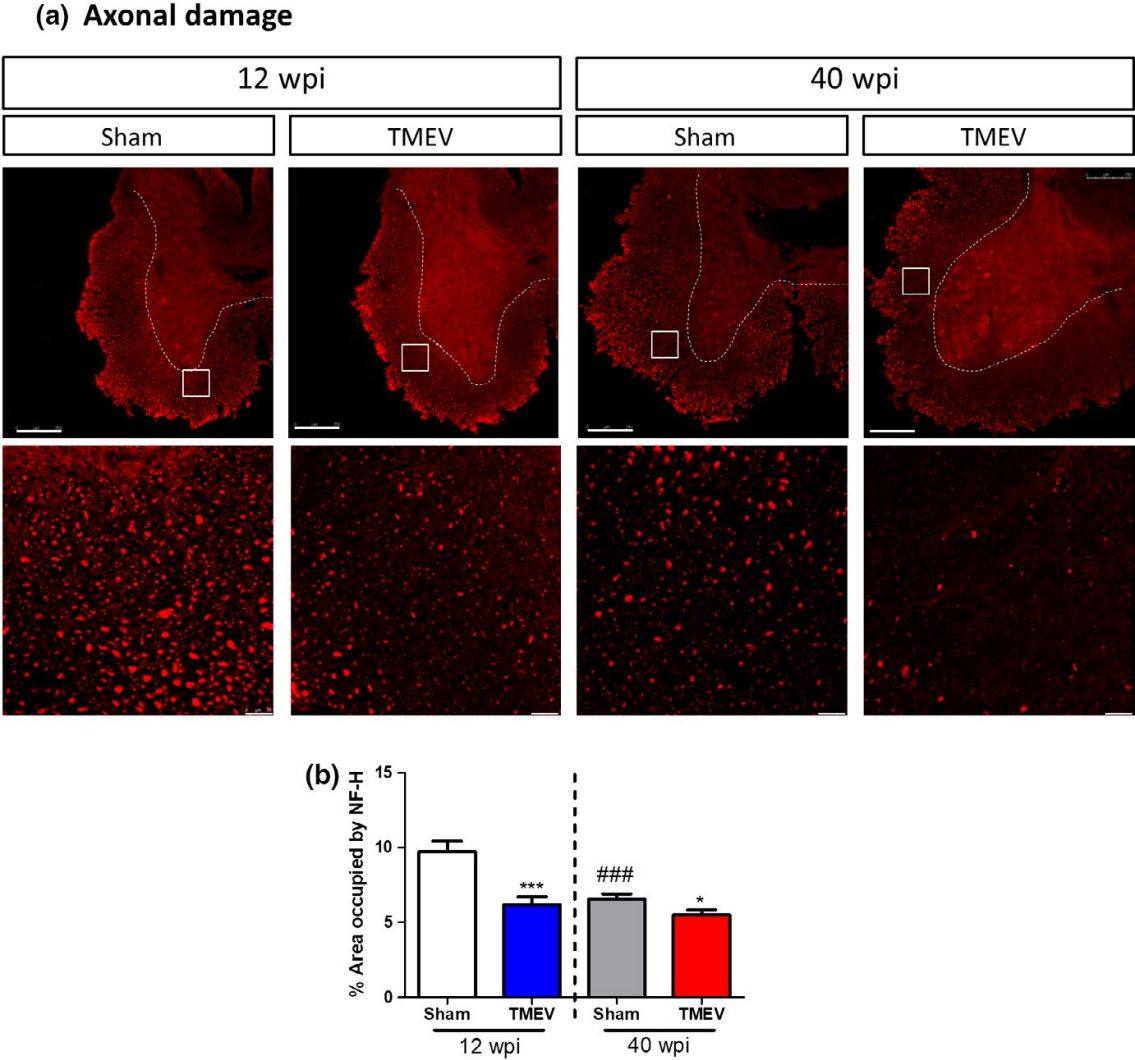
Aging increases axonal damage. (a) Representative confocal images of transverse ventral spinal cord sections at cervical level immunostained for the heavy chain of Neurofilament (NF‐H). Scale bar 250μm. Higher magnification images from the square marked area (63×) are presented below. Scale bar 25 μm. (b) Quantification of the area stained by the NF‐H antibody, expressing the data as the mean ± SEM (*n* = 3–5 mice for each group). Statistics: *p < 0.05 vs. sham at the same age; ***p < 0.001 vs. sham at the same age; ###*p* < 0.001 vs. the same experimental group at 12 wpi

As axon damage in the spinal cord is a well‐established hallmark of TMEV‐IDD (Ure & Rodriguez, 2000), next we evaluated the integrity of axons in transverse sections of the cervical spinal cord of young and old mice by immunolabeling with the NF‐H antibody (Figure 4a). In agreement with our previous work (Feliú et al., 2017), 12 weeks after intracranial viral inoculation there is weaker NF‐H immunostaining in the ventral spinal cord at cervical level, which reflects increased axonal damage (*p* < 0.001; Figure 4b). The loss of axon integrity was higher in old TMEV mice (*p* < 0.05 vs. young TMEV Figure 4b). Surprisingly, this weaker NF‐H labeling was also found in the spinal cord of old sham mice (*p* < 0.001 vs. young sham), although it was more marked in their counterpart mice infected with the virus.

### 2.5 Motor disability in young and old mice infected with Theiler's virus

**FIGURE 5:**
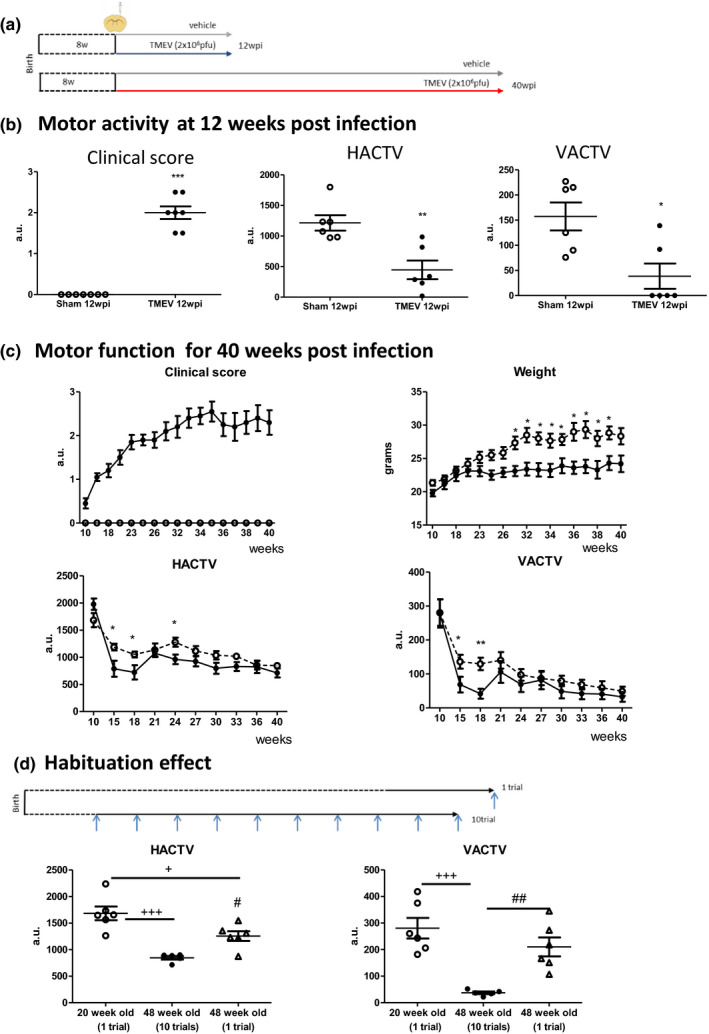
Progression of clinical score and motor activity changes with age. (a) Scheme of the experimental groups where 8‐week‐old mice were infected with 2 x 10^6^ pfu of Theiler's virus and followed for 12 or 40 weeks. (b) Horizontal (HACTV) and vertical (VACTV) motor activity were evaluated for 10 min in the Activity cage at 12 wpi. The clinical score was also evaluated. (c) Clinical score, weight, and Horizontal (HACTV) and Vertical (VACTV) activities were measured once a week from 10 to 40 wpi. (d) Habituation to the Activity cage was evaluated by comparing the motor activity of 20‐week‐old mice (one trial), 48‐week‐old mice (one trial), and 48‐week‐old mice (10 trials). The data represent the mean ±SEM: *p < 0.05 vs. sham at the same age; **p < 0.01 vs. sham at the same age; ^+^ *p* < 0.05 vs. 20‐week‐old mice; ^+++^ *p* < 0.001 vs. 20‐week‐old mice; ^#^ *p* < 0.05 vs. 48‐week‐old mice (10 trials); ^##^ *p* < 0.01 vs. 48‐week‐old mice (10 trials). Number of mice: *n* = 6 sham 12 wpi; *n* = 10 TMEV 12 wpi; *n* = 13 sham 40 wpi; *n* = 13 TMEV 40 wpi. TMEV, Theiler's murine encephalomyelitis virus

Given the changes in innate and adaptive immunity between young (12 wpi) and elderly (40 wpi) TMEV‐infected mice, we further assessed their motor deficits (see scheme in Figure 5a). Then, we evaluated the evolution of the clinical score, the weight, and the spontaneous horizontal and vertical activities (HACTV and VACTV, respectively) over the course of the disease. As established (Mecha et al., 2013; Wootla et al., 2016) mice suffered notable motor deficits 12 weeks after TMEV infection, with a reduction in both HACTV (*p* < 0.01 vs. sham 12 wpi) and VACTV (*p* < 0.05 vs. sham 12 wpi), together with an increase in their clinical score (*p* < 0.001: Figure 5b). The progressive course of the disease was confirmed by the slowly progressive clinical score until week 35; then, there was a stabilization of the severity score until week 40. Two TMEV mice died during this stabilization period of time. The group of TMEV‐infected mice showed lost weight throughout the course of the disease (*p* < 0.05 vs. sham mice). We also followed the motor performance of the mice until the 40th wpi (Figure 5c). A clear habituation profile to the context of the activity cage was observed for the HACTV and VACTV in both sham and TMEV mice from 21 to 40 wpi. Indeed, similar age mice that performed 10 trials showed less HACTV (*p* < 0.05) and VACTV (*p* < 0.01) than those performed only one trial (Figure 5d). Although habituation to the cage (lack of novelty) may influence the results obtained, we cannot rule out that sham mice become less active as they age at least in deambulatory activity (*p* < 0.05 one trial 20 weeks old vs. 48 weeks old, Figure 5D).

### 2.6 Aging increases Treg cells and effector memory CD4^+^ and CD8^+^ T cells in the spleen of TMEV‐infected mice

**FIGURE 6:**
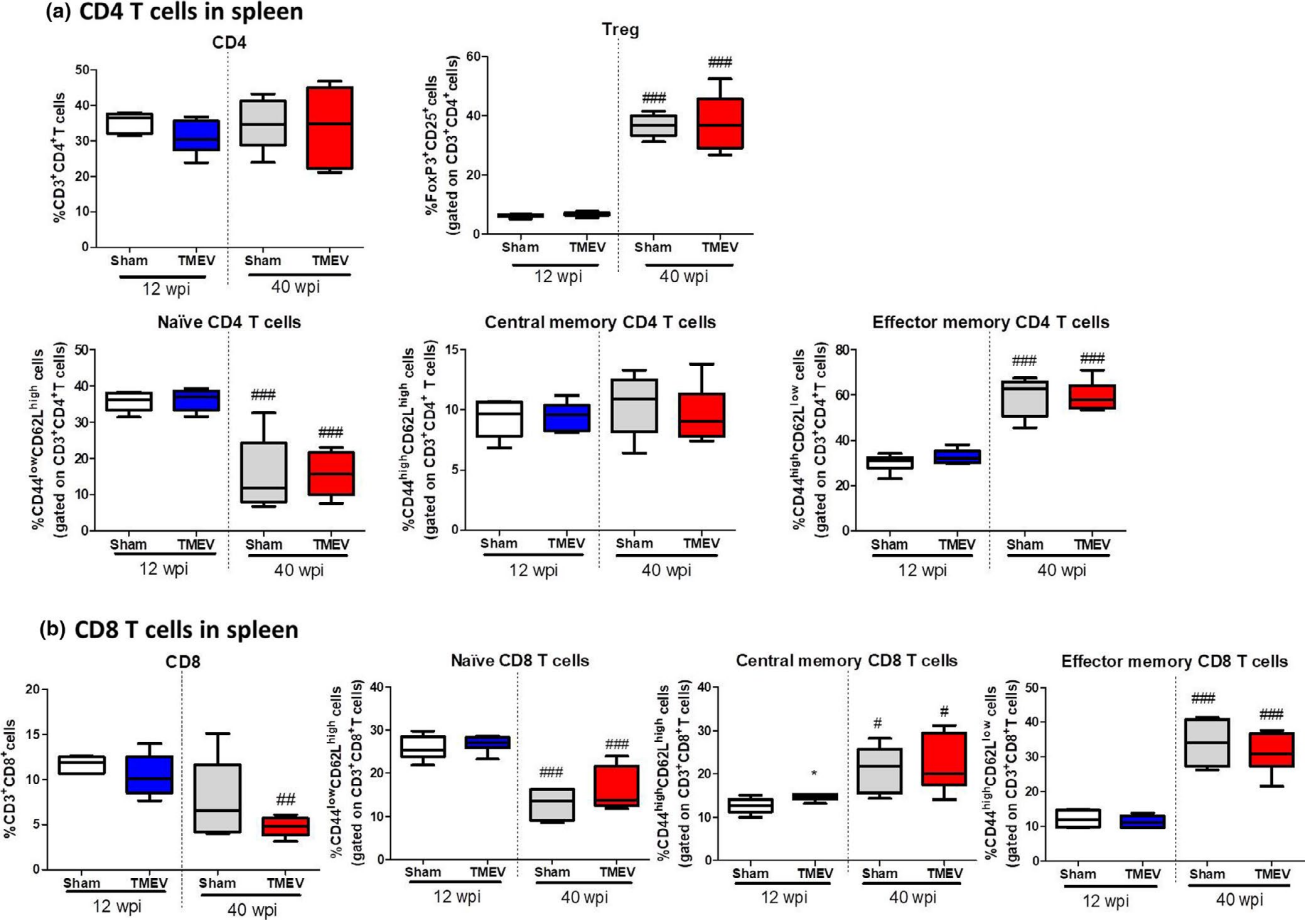
Changes in the peripheral T‐cell response associated with aging. A spleen cell suspension was stained to analyze the different CD4^+^ and CD8^+^ T‐cell subpopulations. (a) Quantification of the percentage of CD3^+^CD4^+^ T cells along with the subpopulations of Treg (Foxp3^+^CD25^+^ cells), Naïve (CD44^low^CD62L^high^), central memory (CD44^high^CD62L^high^), and effector memory (CD44^high^CD62L^low^) CD4^+^ T cells. (b) Quantification of the proportion of CD3^+^CD8^+^ T cells along with the subpopulations of Naïve (CD44^low^CD62L^high^), central memory (CD44^high^CD62L^high^), and effector memory (CD44^high^CD62L^low^) CD8^+^ T cells. The data are expressed as the mean ± SEM:*p < 0.05 vs. sham at the same age; #*p* < 0.05 vs. the same experimental group at 12 wpi; ##*p* < 0.01 vs. the same experimental group at 12 wpi; ###*p* < 0.001 vs. the same experimental group at 12 wpi (*n* = 6 sham 12 wpi; *n* = 10 TMEV 12 wpi; *n* = 7 sham 40 wpi; *n* = 7 TMEV 40 wpi). TMEV, Theiler's murine encephalomyelitis virus

The peripheral immune cell changes were modest in the TMEV‐IDD model in accordance with our previous studies (Carrillo‐Salinas et al., 2017). In the spleen, there were no changes in the percentage of CD4^+^ T cells between sham and TMEV mice irrespective of their age (Figure 6b). However, there was an age‐related increase in the percentage of Tregs in both old sham and TMEV mice (*p* < 0.001 vs. 12 wpi sham or 12 wpi TMEV mice: Figure 6b). An increase in the levels of Tregs suggests that old SJL/J mice may exhibit an exacerbated suppression of the peripheral inflammatory responses compromising CD4^+^ T cells response to pathogens. Furthermore, the percentage of naïve CD4^+^ T cells was reduced in sham and TMEV old mice in comparison with their corresponding younger mice (*p* < 0.001 vs. 12 wpi sham or 12 wpi TMEV mice: Figure 6a). This reduction was compensated by an increase in effector memory CD4^+^ T cells (*p* < 0.001 vs. young sham or TMEV mice: Figure 6a) without any change in the proportion of central memory CD4^+^ T cells. Regarding CD8^+^ T cells while TMEV infection did not substantially alter their percentage in the spleen of young mice, they were significantly diminished in old TMEV mice (*p* < 0.01 vs. 12 wpi TMEV mice: Figure 6b). Interestingly, old sham and TMEV mice have a lower proportion of naïve CD8^+^ T cells, with an increase in central memory (*p* < 0.05 vs. young sham and TMEV mice: Figure 6b) and effector memory CD8^+^ T cells (*p* < 0.001 vs. 12 wpi sham or TMEV mice: Figure 6b).

### 2.7 Aging‐associated spleen innate immune response in the TMEV‐IDD model involves fewer monocytes/macrophages and increased PDL‐1 expression

**FIGURE 7:**
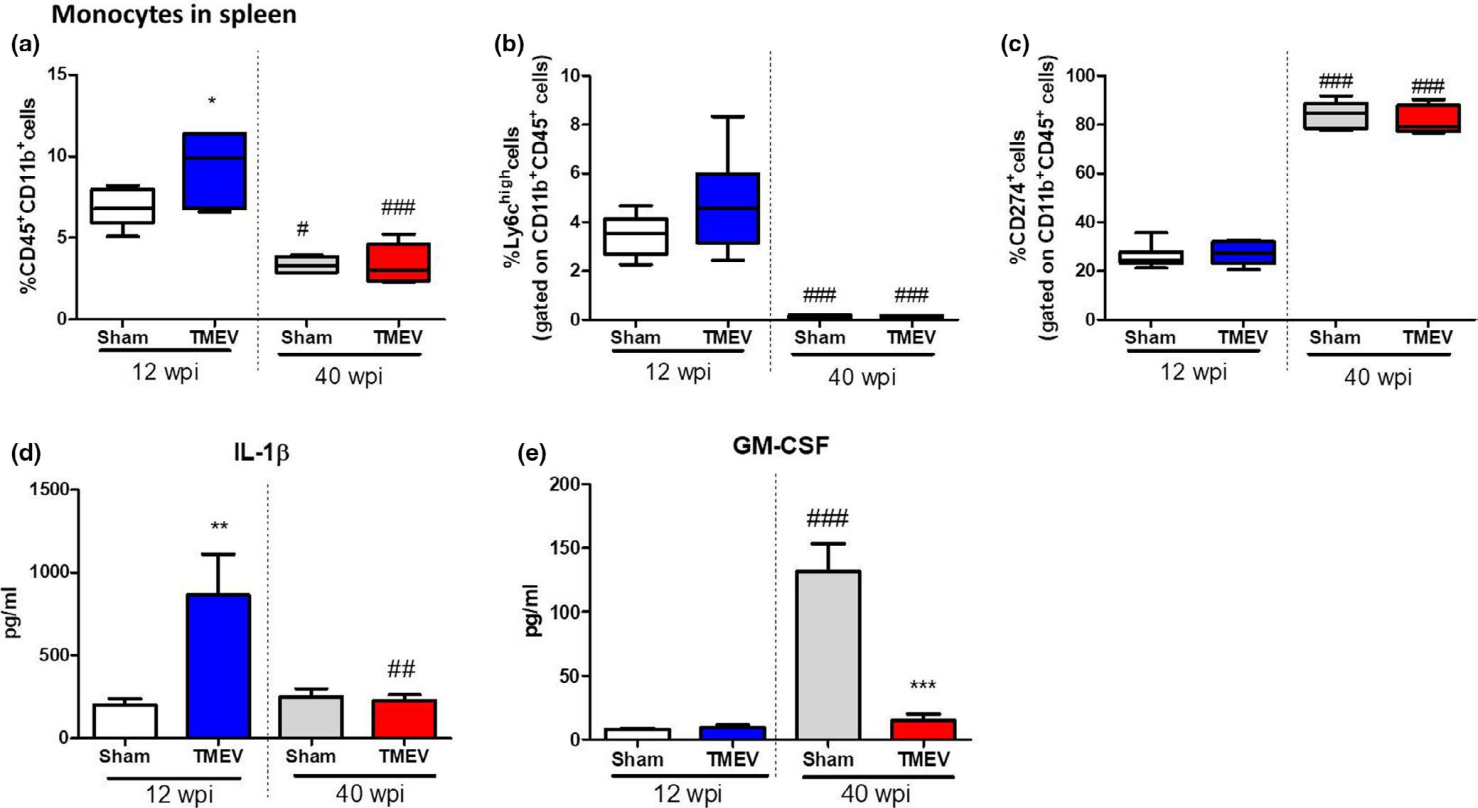
The peripheral innate immune response was dampened by aging. (a) Quantification of the percentage of CD45^+^CD11b^+^ cells in the spleen. (b, c) Quantification of the percentage of Ly6c^high^ or CD274^+^ cells, respectively, gated on CD45^+^CD11b^+^ monocyte cells. (d) CD11b^+^ monocyte cells isolated using Miltenyi beads were stimulated with LPS and IL‐1β production was measured by ELISA. (e) Splenocyte cell suspension was stimulated as indicated in the Materials and Methods, and GM‐CSF production was measured by ELISA on the supernatant. The data are expressed as the mean ± SEM (*n* = 5 mice for each group): *p < 0.05 vs. sham at the same age; **p < 0.01 vs. sham at the same age; ***p < 0.001 vs. sham at the same age; ^#^ *p* < 0.05 vs. the same experimental group at 12 wpi; ^##^ *p* < 0.01 vs. the same experimental group at 12 wpi; ^###^ *p* < 0.001 vs. the same experimental group at 12 wpi

Age‐related changes in the innate immune responses were observed in the spleen of TMEV mice (Figure 7). Most significantly, there were more monocytes/macrophages at 12 wpi in TMEV mice (*p* < 0.05 vs. 12 wpi sham mice: Figure 7a) but not at 40 wpi, the latter having a smaller proportion of macrophages (*p* < 0.001 vs. 12 wpi TMEV mice: Figure 7a). Old sham mice also display reduced monocytes frequency (*p* < 0.05 vs. 12 wpi sham mice: Figure 7A). Intriguingly, the proportion of Ly6C^high^ CD45^+^ CD11b^+^ monocytes was diminished in both, old sham and TMEV mice (*p* < 0.001 vs. 12 wpi sham or 12wpi TMEV mice: Figure 7b), suggesting a reduced ability of innate immune response against pathogens in old subjects. The opposite occurred with CD274^+^ CD45^+^ CD11b^+^ monocyte frequency since old sham and TMEV mice exhibited a higher percentage of this type of cell expressing PDL‐1^+^ (*p* < 0.001 vs. 12 week pi sham or TMEV mice: Figure 7c). Furthermore, *ex vivo* functional studies indicated a lack of IL‐1β responsiveness to LPS in spleen monocytes purified from old TMEV mice (Figure 7d). One significant and striking finding was the elevated levels of GM‐CSF in *ex vivo* splenocytes isolated from old sham mice and stimulated with Phorbol 12‐myristate 13 acetate (PMA)/ionomycin (*p* < 0.001 vs. 12 wpi sham mice: Figure 7e), while their counterparts isolated from old TMEV mice maintained similar low amount of GM‐CSF as splenocytes isolated from young 12 wpi TMEV mice.

## 3 DISCUSSION

Although multiple alterations at the cellular level contribute to the transition to progressive forms of MS the underlying changes in the CNS compartment remain to be determined. TMEV‐IDD model features a chronic progressive disease course which lasts for the entire lifespan in susceptible mice showing a dynamic of motor dysfunction and score severity that depends on the virus strain (McCarthy et al., 2012). In the viral model used here, the brain cellular remodeling that occurs in adaptive immunity as mice get old reflects a blunted TMEV immune response, being largely a consequence of the changes observed in the old control mice that exhibited an anomalous presence of CD4^+^ and CD8^+^ T cells. Aging also reduces the naïve and central memory T cells, while effector memory CD4^+^ T cells were increased in the CNS of old control mice as described in the periphery in the human aging (Ventura et al., 2017). There is increased interest in the contribution of CD8^+^ T cells as effectors of the pathological immune reaction that damage the CNS in MS and its animal models. CD8^+^ T cells are the most prevailing lymphocyte population in early MS lesions but they may remain in the brain and spinal cord as tissue‐resident cells propagating focal neuroinflammation associated with aging (Lassmann, 2019). Though we could not establish a direct causal proof between the changes in CD8^+^ T cells and the extent of axonal loss integrity, studies on the TMEV model suggest that CD8^+^ T cells may directly injure the demyelinated axons (Johnson et al., 2001; Deb et al., 2010. It remains unclear why there is an increased frequency of CD8^+^ T cells in the CNS of old control mice but we suggest that this event would be related to the increased axonal damage that we found in these mice when compared with their counterpart young mice. In recent years, aging has been associated with an increase of leukocyte recruitment to the CNS in normal mice, primarily conventional CD8^+^ T cells (Ritzel et al., 2016). Indeed, studies on donor human brain tissue identified an enrichment of resident CD8^+^ T cells in white matter and in regions where the BBB becomes leaky, particularly in the SVZ (Moreno‐Valladares et al., 2020). The above findings suggest that leukocyte recruitment in the aging mouse brain may have translational relevance. In particular, CD8^+^ T‐cell accumulation in older humans has been suggested to be largely driven by ongoing sub‐clinical responses to persistent viruses like cytomegalovirus (Nikolich‐Žugich et al., 2020).

Furthermore, age‐related changes in B cells are contributing to CNS immunosenescence (Cancro, 2020). In MS, B‐cell infiltrates are associated with the presence of oligoclonal IgG bands in the CSF, a diagnostic hallmark in 95% of patients due to intrathecal antibody production in the CNS compartment (Villar et al., 2014). However, the exact mechanisms regulating B‐cell accumulation and their persistence in the CNS remain elusive, particularly in progressive MS. Importantly, in the TMEV‐IDD model antibody synthesis in the CNS was similar to than observed in MS (Pachner et al., 2011). The increased frequency of B cells in old control mice is particularly intriguing but it may be related to higher levels of antibodies in the CNS as described also in the human elderly (Biragyn et al., 2017). As in the case of T cells, the increased counts of B cells in old sham and old TMEV mice put forward that aging favors CNS compartmentalized cellular and humoral immune responses. A prominent involvement of B cells in the CNS compartment has been seen previously during TMEV‐IDD, including robust intrathecal antibody (ItAb) production and even aggregates within the meningeal and perivascular regions (DiSano et al., 2019). Indeed, a shift in trafficking profile of B cells from the periphery to the brain during chronic TMEV‐IDD has been proposed (Navarrete‐Talloni et al., 2010) in agreement with the relevance of CNS compartment in progressive MS. We provide here new evidence about the aging‐associated decline on the percentage of CD5^+^CD1d^high^ Breg cells in old control and TMEV mice. Nevertheless, we cannot infer the relevance of Bregs reduction in the specific neurodegenerative process of TMEV‐IDD as it was also observed under non‐pathological condition (old sham mice).

The neuroinflammatory component of MS and its animal models preferentially involves microglia/macrophage cells associated with cytotoxic mechanisms, but also with endogenous tissue repair (Liu et al., 2018; Mecha et al., 2016). Although the purinergic receptor P2RY12 labels homeostatic microglia as its expression is downregulated when microglia is activated (Zrzavy et al., 2017) several studies suggest that P2RY12 also identified other populations of microglia not only homeostatic microglia (Walker et al., 2020). Here, we employed different gating strategies by FACs technology trying to identify microglia by considering CD45^+^P2YR12^+^ or CD45^low^CD11b^+^ cells. However, the limitations of this technique not allow us to discard that microglia losing P2YR12 expression or highly activated microglia upregulating CD45 might be confused as macrophage. In the present study, the frequency of microglial cells labeled with P2RY12 is augmented in young TMEV‐infected mice yet this microglial response appears to be dampened in aging TMEV mice, likely due to the fact that aged control mice showed an elevated percentage of microglia perhaps reflecting an altered microglia turnover (Askew et al., 2017; Damani et al., 2011). When we analyzed the morphological phenotype of aged microglia labeled with Iba‐1, the fact of observing fewer cells in TMEV old mice maintaining the same percentage of Iba‐1 occupied area would suggest a microglia shift toward a big cell body and thick branches, but we cannot discard that macrophages can contribute to the Iba‐1 occupied area as this marker also recognizes macrophages. This shift in microglia may unveil their diminished capacity to mount a normal response to the axon loss that took place in TMEV‐IDD potentially contributing to an accelerated neurodegenerative process. Reduced migration and phagocytosis ability are characteristics of aged microglia that in the context of MS delays remyelination as a consequence of the impaired clearance of myelin debris (Neumann et al., 2009). We guess that microglia and in general, myeloid cells reprograming is context and time dependent and so, a strict definition of microglia markers may result incorrect.

In the present study, we observed that macrophages behave distinctly to microglia in the aging CNS. First, contrary to microglia macrophage frequency did not differ between young and old sham or TMEV mice. Second, the immune checkpoint PDL‐1 expressed by microglia and macrophages show opposite responses. Although the potential contribution of increased microglia expressing PDL‐1 to the age‐associated neurodegeneration in TMEV‐IDD remains unclear, our findings reinforce the interest of PDL‐1 in the CNS compartmentalized immune responses in our model and in progressive MS (Bar‐Or & Antel, 2016). A previous study showed induced expression of PDL‐1 by microglia and macrophages within the CNS, yet not in the periphery shortly after TMEV inoculation (Jin et al., 2013). However, we found increased PDL‐1 expression in spleen macrophages of old control and TMEV mice that is, long time after virus administration. The finding of diminished spleen proportion of Ly6C^high^ CD45^+^ CD11b^+^ monocytes in both control and TMEV mice may uncover a reduced ability of innate immune response against pathogens in the elderly. Besides, our *ex vivo* functional studies show that spleen purified macrophages from TMEV old mice produce less IL‐1β in response to LPS than purified young cells in agreement with that observed in microglial cells (Njie et al., 2012). GM‐CSF is a cytokine that exists at low levels at steady state but increases drastically during infection and inflammation (Shiomi & Usui, 2015). Then, it is difficult to explain the increased GM‐CSF production only by ex vivo splenocytes from old sham mice stimulated with PMA/ionomycin. It is possible that GM‐CSF may be contributing to the increased migration of leukocytes into the CNS that we found elevated in old control mice. Our study has unveiled the impact of aging in cell immune remodeling in control and TMEV diseased mice but has important mechanistic limitations. It should be emphasized that it is not yet clear why leukocytes accumulate in the CNS of aging mice.

Collectively, the results of the present study point out to a key role of neuroinflammation associated with physiological aging and pathological conditions such as TMEV infection. In the TMEV‐IDD model, mice largely display alterations in the innate and adaptive cell immune responses in the CNS. This age‐associated immune dysfunction reduces the ability of the immune system to respond efficiently to the pathological consequences induced by TMEV during the later chronic phases of the disease. The impaired immune response includes T and B cells, microglia, and macrophages emphasizing the new data about Bregs and PDL‐1 expressing microglia. A CNS compartmentalized aging effect was mainly observed although cellular immune alterations occur at the spleen level too. The findings of the present study provide new evidence on the aging‐related changes in immune cell population in the context of a viral progressive model of MS and suggest the need of innovative pharmacological strategies for elderly MS patients.

## 4 EXPERIMENTAL PROCEDURES

### 4.1 Animals and TMEV infection

Six to eight‐week‐old susceptible female SJL/J mice (Charles River; Barcelona, Spain) were maintained under controlled conditions at the Instituto Cajal (CSIC; Madrid, Spain): 12 h light/dark cycle, temperature at 20°C (±2°C) and 40%–50% relative humidity, with ad libitum access to food and water. We used a viral model that resembles the course of progressive MS forms. Viral infection is made at the window of 6–8 weeks age, then, there is latency period to the onset of clinical signs in susceptible strain of mice which show during the chronic phase a slowly progressive disability, with demyelination, neuroinflammation, and axonal damage. The latency period and severity of disease are depending on the strain of virus and its dose (McCarthy et al., 2012). Susceptible SJL/L mice lacked an innate immune population, the natural killer dendritic cell, which play a critical role in early CNS virus clearance as occurs in resistent strain of mice (Chastain et al., 2015). The mice were inoculated in the right brain hemisphere with 2 × 10^6^ plaque‐forming units (pfu) of the Daniel (DA) strain of TMEV, a picornavirus positive‐sense single‐stranded RNA, diluted in 30 μl DMEM supplemented with 10% of FCS, as described previously (Arévalo‐Martín et al., 2003). Sham mice received 30 μl of DMEM supplemented with 10% of FCS. Two time lines of experimental groups were defined: (i) 8‐week‐old infected mice sacrificed 12 weeks post‐infection (wpi: sham *n* = 7, TMEV *n* = 7) and (ii) 8‐week‐old infected mice sacrificed 40 wpi (sham *n* = 11, TMEV=15). Note that the SJL/J strain of mice undergoes physiological aging earlier than other strains of mice, such as C57BL/6 or BALBc (Liu et al., 2014). Specifically, SJL/J mice have a shorter lifespan, with males living 16 months on average and females only 14 months. General health conditions (weight and clinical score) were evaluated periodically every week from 10th to 40th week pi. Clinical scores (Murray et al., 1998) were assigned based on the general appearance and activity of the mice: score 1, mice with a waddling gait; Score 2, mice adopting a more severe waddling gait; score 3, mice that had lost their righting ability, in conjunction with hind limb spasticity; score 4, mice suffering paralysis of their hind limbs; and score 5, mice that were moribund.

All experiments were performed in strict accordance with EU (Directive 2010/63/EU) and Spanish guidelines (Royal Decree 53/2013 BOE no. 34 and Comunidad de Madrid ES 280790000184) following the 3R principles: reduction, refinement, and replacement required by our Ethics Committee. The Ethics Committee on Animal Experimentation at the Instituto Cajal (CSIC) approved all the procedures described in this study (protocol no. 2013/03 CEEA‐IC).

### 4.2 Evaluation of motor function

Theiler's murine encephalomyelitis virus‐induced demyelinating disease is a model of chronic progressive MS in which virus inoculation is followed by a latent period of approximately 12 weeks until the clinical signs and motor deficits appear. The screening of locomotor activity was performed in an activity cage coupled to a Digiscan Analyser (Activity Monitor System; Omnitech Electronics Inc.). Horizontal (HACTV) and Vertical (VACTV) activity were evaluated through the total number of horizontal and vertical sensor beam interruptions in a 10 min session. HACTV and VACTV activities were measured once a week after infection (wpi) from 10 to 40 wpi.

### 4.3 Sample collection

The mice were anesthetized with pentobarbital (Doletal, 50 mg/kg body weight), and their spleen was removed, kept in cold Roswell Park Memorial Institute (RPMI), and processed for flow cytometry. After transcardial perfusion with saline (0.9% NaCl), the brain and spinal cord were removed and processed for flow cytometry as indicated below. A little segment of the cervical spinal cord from at least 3 mice per experimental group was reserved for immunohistochemical analysis.

### 4.4 Flow cytometry Antibodies

A CNS and spleen leukocyte suspension was obtained as described previously (Carrillo‐Salinas et al., 2017). Isolated cells were incubated with anti‐CD16/CD32 (Affymetrix Inc.) for FcR blockade and labeled with anti‐mouse antibodies: PE‐conjugated anti‐CD44 (2.4 μg/ml), PE‐conjugated anti‐CD274 (2.4 μg/ml), PerCP‐Cy5.5‐conjugated anti‐CD4 (1.2 μg/ml), PECy7‐conjugated anti‐Ly6c (1.2 μg/ml), APC‐Cy7‐conjugated anti‐CD11b (1.2 μg/ml), APC‐conjugated anti‐CD62L (2.4 μg/ml), APC‐conjugated anti‐CD25 (2.5 μg/ml), and APC‐conjugated anti‐CD1d (2.4 μg/ml; all from eBioscience); APC‐conjugated anti‐P2yR12 (2.5 μg/ml) and APC‐Cy7‐conjugated anti‐CD3 (1.2 μg/ml) (both from Biolegend); PE‐conjugated anti‐CD5 (2.2 μg/ml), PerCP‐Cy5.5‐conjugated anti‐B220 (2.4 μg/ml), PerCP‐Cy5.5‐conjugated anti‐CD45 (1.2 μg/ml), PECy7‐conjugated anti‐CD8 (1.2 μg/ml) and PECy7‐conjugated anti‐CD19 (2.4 μg/ml; all from BD Pharmingen). The cells were fixed for 30 min with fixation buffer (Affymetrix Inc.). For FoxP3 detection, the cells were suspended in Fixation/Permeabilization buffer for 30 mins prior to staining with an anti‐FoxP3 antibody (3 μg/ml; BD Pharmingen). At least 50,000 events were registered in each experiment on a FACSAria flow cytometer (BD Biosciences), excluding duplets from the analysis. The data were analyzed using FACSDiva analysis software (BD Biosciences).

### 4.5 Immunohistochemistry

A fragment of the cervical spinal cord was fixed in 4% paraformaldehyde prepared in 0.1 M phosphate buffer (PB), cryoprotected in a 30% solution of sucrose in 0.1 M PB and frozen at −80°C. Free‐floating, transverse cryostat sections (30 μM) were rinsed in 0.1 M PB, blocked in 5% Normal Goat serum in PB 0.1 M and incubated overnight at 4°C with the Iba‐1 (ionized calcium‐binding adaptor molecule 1, diluted 1:1000: Wako Chemical Pure Industry) or NF‐H antibody (Neurofilament H 1:1000: Millipore). After washing, the sections were incubated for 1h with an Alexa‐fluor 594 conjugated goat anti‐rabbit antibody (1:1000), washed and mounted with Mowiol. Based on our previous experience with the antibodies used in the present study, the specificity of the staining was confirmed by omitting the primary antibody and not by a matching isotype control.

### 4.6 Microscopy and image analysis

Immunofluorescence images were acquired on a Leica TCS SP5 confocal microscope and immunohistochemical staining was assessed with a Zeiss Axiocam high‐resolution digital color camera. Individual optical sections were examined by analyzing five to six sections (ventral spinal cord area) from at least three to five animals per group. Staining was quantified using Image J software (NIH), maintaining the threshold intensity constant to compare the experimental and control images obtained within the experiments.

### 4.7 Monocyte and Splenocyte ex vivo experiments

Spleen monocytes were isolated using a commercial cell isolation kit following the manufacturer's instructions (Miltenyi; Biotec Inc.). Cells from each animal were plated (10^6^ cells/well) and stimulated for 5 h with LPS (1 μg/ml). The cell medium was then collected, centrifuged, and the supernatant frozen. Splenocytes from each mice were plated (10^6^ cells/well) and cultured in the medium RPMI supplemented with immobilized hamster anti‐mouse CD3ε (10 μg/ml: BD biosciences) and hamster anti‐mouse CD28 (2 μg/ml: BD biosciences). Two days later, the cells were cultured for another three days in RPMI plus IL‐2 (20 ng/ml) and IL‐4 (50 ng/ml: Preprotech), and then stimulated for 5 h with PMA (20 ng/ml) /ionomycin (1 μg/ml). The cell medium was collected centrifuged and the supernatant frozen.

### 4.8 ELISAs

The production of IL‐1β by monocytes and of GM‐CSF (granulocyte‐macrophage colony‐stimulating factor) by splenocytes from each mice was measured by solid‐phase sandwich ELISA using Quantikine kits (R&D systems Inc.), following the manufacturer's instructions. The assay's sensitivity was 1.8 pg/ml for GM‐CSF and 2.31 pg/ml for IL‐1β.

### 4.9 Statistical analysis

The data were expressed as the mean ± SEM, and they were analyzed using GraphPath Prism5 Software. One‐way ANOVA followed by the Bonferroni post hoc test was used to determine the statistical significance. For non‐parametric analyses, a Kruskal–Wallis and Mann–Whitney U test were applied. Multiple data of motor activity obtained by the repeated measurement from one subject were analyzed by ANOVA for repeated measures. *p* < 0.05 were consider significant.

## CONFLICTS OF INTEREST

The author(s) have no potential conflicts of interest.

## AUTHOR CONTRIBUTIONS

Leyre Mestre and Carmen Guaza conceived and supervised the study. Graciela Alonso and Ana Feliú conducted the experimental procedures. Miriam Mecha and Carolina Martín performed immunohistochemistry. Leyre Mestre analyzed the data. Leyre Mestre and Carmen Guaza wrote the manuscript with input from Luisa M. Villar. All authors approved the final version of the manuscript.

## Data Availability

Data available on request from the authors.
